# Potassium (1-methoxy­carbonyl-2-methyl­prop-2-en-2-yl­idene)azinate

**DOI:** 10.1107/S1600536810010159

**Published:** 2010-03-27

**Authors:** Cédric Reuter, Jörg M. Neudörfl, Hans-Günther Schmalz

**Affiliations:** a Department für Chemie der Universität zu Köln, Greinstrasse 4, 50939 Köln, Germany

## Abstract

In the title compound, K^+^·C_6_H_8_NO_4_^−^, the K^+^ cations have a coordination number of seven and are surrounded by four bidentate azinate anions. The methyl­ene groups of the anions are always directed towards the coordinated potassium cations. The N—C—C—C torsion angle is 101.2 (2)°. The orthogonal non-conjugated nature of the salt confirms the supposed geometry and reactivity of this compound.

## Related literature

For a short overview of peptidomimetics, see: Grauer *et al.* (2009 ▶); Vagner *et al.* (2008 ▶); Wu *et al.* (2008 ▶). For the synthesis of peptidomimetics, amino-acid-based building blocks play a key role in the assembly of these structures, see: Kemp, Boyd & Muendel (1991 ▶); Kemp, Curran *et al.* (1991 ▶); Beal *et al.* (2000 ▶); Kühne *et al.* (2008 ▶). A known deprotonation/proton­ation sequence (Bouveault & Wahl, 1901 ▶) was used in the synthesis of the title compound. The protonation of the title compound occurs exclusively at the α-position and no proton­ation of the methyl­ene group was observed (Baldwin *et al.*, 1977 ▶). 
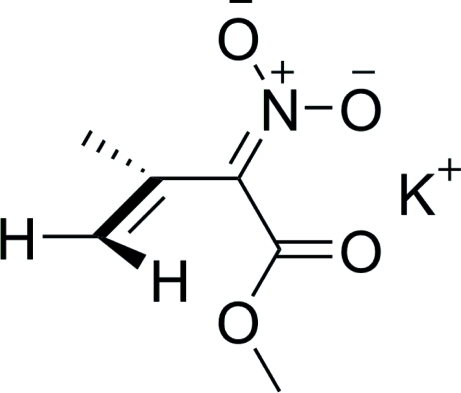


## Experimental

### 

#### Crystal data

K^+^·C_6_H_8_NO_4_^−^
*M* *_r_* = 197.23
Monoclinic, 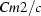

*a* = 23.9269 (13) Å
*b* = 5.2909 (2) Å
*c* = 14.2510 (7) Å
β = 113.361 (2)°
*V* = 1656.21 (14) Å^3^
*Z* = 8
Mo *K*α radiation
μ = 0.62 mm^−1^
*T* = 100 K
0.20 × 0.15 × 0.03 mm

#### Data collection

Nonius KappaCCD diffractometer
6264 measured reflections
1810 independent reflections
1416 reflections with *I* > 2σ(*I*)
*R*_int_ = 0.041

#### Refinement

*R*[*F* ^2^ > 2σ(*F* ^2^)] = 0.028
*wR*(*F* ^2^) = 0.061
*S* = 1.01
1810 reflections
111 parameters
H-atom parameters constrained
Δρ_max_ = 0.31 e Å^−3^
Δρ_min_ = −0.27 e Å^−3^

### 

Data collection: *COLLECT* (Hooft, 1998 ▶); cell refinement: *DENZO* (Otwinowski & Minor, 1997 ▶); data reduction: *DENZO*; program(s) used to solve structure: *SHELXS97* (Sheldrick, 2008 ▶); program(s) used to refine structure: *SHELXL97* (Sheldrick, 2008 ▶); molecular graphics: *SCHAKAL99* (Keller, 1999 ▶); software used to prepare material for publication: *PLATON* (Spek, 2009 ▶), *publCIF* (Westrip, 2010 ▶) and *ORTEP* (Davenport *et al.*, 1999 ▶).

## Supplementary Material

Crystal structure: contains datablocks global, I. DOI: 10.1107/S1600536810010159/jj2024sup1.cif

Structure factors: contains datablocks I. DOI: 10.1107/S1600536810010159/jj2024Isup2.hkl

Additional supplementary materials:  crystallographic information; 3D view; checkCIF report

## Figures and Tables

**Table 1:** Selected bond lengths (Å)

K1—O1^i^	2.7036 (10)
K1—O2^ii^	2.7539 (11)
K1—O3^i^	2.7988 (11)
K1—O1	2.7994 (11)
K1—O2	2.8896 (10)
K1—O3^iii^	2.8970 (12)
K1—O1^iii^	2.9080 (11)

Symmetry codes: (i) 
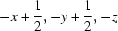
; (ii) 
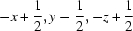
; (iii) 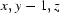.

**Fig. 1.**
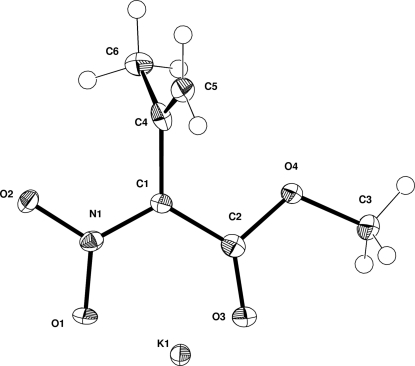
A view of (I). Displacement ellipsoids are drawn at the 50% probability level.

**Fig. 2.**
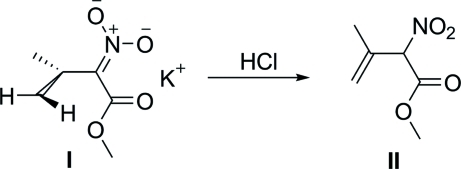
Protonation of the title compound (I).

**Fig. 3.**
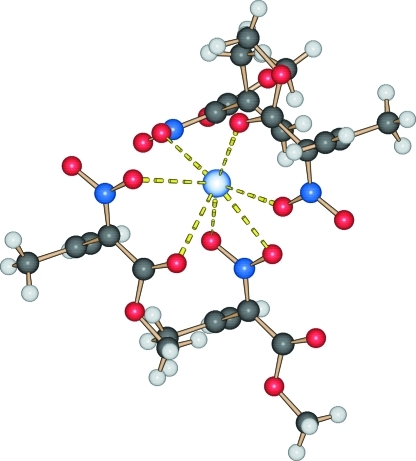
Coordination sphere of an isolated potassium Cation.

**Fig. 4.**
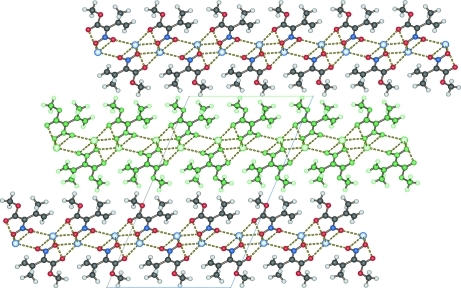
View of the unit cell along the b-axis.

## supplementary crystallographic information

### Comment

In the last decade, interest in peptidomimetics has lead to a fast growing research field within organic chemistry (Grauer *et al.*, 2009). Artificial peptide-like compounds are used to explore the principles of protein-protein interactions and their modulation (Vagner *et al.*, 2008; Wu *et al.*, 2008). In the synthesis of different peptidomimetics, amino acid based building blocks play a key role in the assembly of these structures (Kemp, Curran *et al.*, 1991; Kemp, Boyd & Muendel, 1991; Beal *et al.*, 2000; Kühne *et al.* 2008). In the context of our work we used an already known deprotonation/protonation sequence (Bouveault *et al.*, 1901) to synthesize our compound. The protonation of the title compound occurs exclusively at the α-position whereas no protonation of the methylene group was observed (Baldwin *et al.*, 1977).

In the title compound, C_6_H_8_KNO_4_, (I),(Fig. 1), the deconjugation within the molecule combined with the high basicity of the nitro enolate provides a convincing explanation for the high selectivity of this reaction (Fig. 2). The crystal structure supports the assumption made by Baldwin *et al.* about the geometry of this potassium salt. The potassium cations in the crystal stucture have a coordination number of seven and are surrounded by four azinate anions with K—O distances from 2.704 (1) to 2.908 (1) Å (Fig. 3). These can either bind via the carbonyl group or the nitrogen-bonded oxygen atoms whereas both motifs can be found as bridging units. The resulting polar layers of potassium cations surrounded by oxygen atoms are perfectly shielded by the methyl and methylene residues (Fig. 4). This results in loose interactions between the different layers and explains the facile mechanical fissility of the crystals.

### Experimental

The title compound, C_6_H_8_KNO_4_, was prepared in good yield from methyl 3-methyl-2-nitrobut-2-enoate by deprotonation with potassium hydride. In a dry, argon-flushed 50 ml flask, 30.1 mmol of potassium hydride where suspended in 15 ml of dry THF. The suspension was cooled to 0 °C and a solution of 30.1 mmol methyl-3-methyl-2-nitrobut-2-enoate in 5 ml of THF was added via syringe over 30 min. After stirring for 5 h at room temperature a small amount of *n*-octanol was added at 0 °C to destroy the excess of potassium hydride. The paste was filtrated, washed three times with THF and dried in vacuo to give 4.950 g (25.1 mmol, 83%) of an ochre powder. A portion of the salt was recrystallized from MeOH/THF to give colourless prisms.

### Refinement

Hydrogen atoms were located in difference Fourier maps and refined at idealized positions (C—H = 0.98 Å for methyl H atoms and 0.95 Å for all other H Atoms) using a riding model. The U values of the hydrogens are constrained relative to U_eq_ of the parent carbon atom (1.2 x U_eq_(C) for C—H_2_ and 1.5 x U_eq_(C) for methyl H).

### Crystal data

**Table:**

K^+^·C_6_H_8_NO_4_^−^	*F*(000) = 816
*M**_r_* = 197.23	*D*_x_ = 1.582 Mg m^−^^3^
Monoclinic, *C**m*2/*c*	Melting point: 180.7(10) K
Hall symbol: -C 2yc	Mo *K*α radiation, λ = 0.71073 Å
*a* = 23.9269 (13) Å	Cell parameters from 6264 reflections
*b* = 5.2909 (2) Å	θ = 1.9–27.0°
*c* = 14.2510 (7) Å	µ = 0.62 mm^−^^1^
β = 113.361 (2)°	*T* = 100 K
*V* = 1656.21 (14) Å^3^	Platlet, colourless
*Z* = 8	0.20 × 0.15 × 0.03 mm

### Data collection

**Table:**

Nonius KappaCCD diffractometer	1416 reflections with *I* > 2σ(*I*)
Radiation source: fine-focus sealed tube	*R*_int_ = 0.041
graphite	θ_max_ = 27.0°, θ_min_ = 1.9°
Phi/ω–Scans scans	*h* = −30→30
6264 measured reflections	*k* = −6→6
1810 independent reflections	*l* = −18→18

### Refinement

**Table:**

Refinement on *F*^2^	Primary atom site location: structure-invariant direct methods
Least-squares matrix: full	Secondary atom site location: difference Fourier map
*R*[*F*^2^ > 2σ(*F*^2^)] = 0.028	Hydrogen site location: inferred from neighbouring sites
*wR*(*F*^2^) = 0.061	H-atom parameters constrained
*S* = 1.01	*w* = 1/[σ^2^(*F*_o_^2^) + (0.0266*P*)^2^] where *P* = (*F*_o_^2^ + 2*F*_c_^2^)/3
1810 reflections	(Δ/σ)_max_ = 0.001
111 parameters	Δρ_max_ = 0.31 e Å^−^^3^
0 restraints	Δρ_min_ = −0.27 e Å^−^^3^

### Special details

**Table:**

Geometry. All esds (except the esd in the dihedral angle between two l.s. planes) are estimated using the full covariance matrix. The cell esds are taken into account individually in the estimation of esds in distances, angles and torsion angles; correlations between esds in cell parameters are only used when they are defined by crystal symmetry. An approximate (isotropic) treatment of cell esds is used for estimating esds involving l.s. planes.
Refinement. Refinement of *F*^2^ against ALL reflections. The weighted *R*-factor wR and goodness of fit *S* are based on *F*^2^, conventional *R*-factors *R* are based on *F*, with *F* set to zero for negative *F*^2^. The threshold expression of *F*^2^ > σ(*F*^2^) is used only for calculating *R*-factors(gt) etc. and is not relevant to the choice of reflections for refinement. *R*-factors based on *F*^2^ are statistically about twice as large as those based on *F*, and *R*- factors based on ALL data will be even larger.

### Fractional atomic coordinates and isotropic or equivalent isotropic displacement parameters (Å^2^)

**Table:**

	*x*	*y*	*z*	*U*_iso_*/*U*_eq_	
K1	0.269013 (15)	−0.06555 (6)	0.11206 (2)	0.01586 (11)	
O1	0.22159 (5)	0.42337 (19)	0.06333 (8)	0.0183 (3)	
O2	0.20272 (5)	0.26997 (19)	0.19103 (8)	0.0172 (2)	
O3	0.15057 (5)	0.81224 (18)	−0.04385 (8)	0.0194 (3)	
O4	0.07200 (5)	0.87761 (19)	0.00265 (8)	0.0191 (3)	
N1	0.19003 (6)	0.4306 (2)	0.11777 (9)	0.0144 (3)	
C1	0.14436 (7)	0.5975 (3)	0.10141 (11)	0.0142 (3)	
C2	0.12553 (7)	0.7664 (3)	0.01413 (12)	0.0155 (3)	
C3	0.04879 (8)	1.0606 (3)	−0.07901 (12)	0.0213 (4)	
H3A	0.0089	1.1211	−0.0844	0.032*	
H3B	0.0446	0.9813	−0.1436	0.032*	
H3C	0.0771	1.2034	−0.0644	0.032*	
C4	0.11349 (7)	0.5958 (3)	0.17409 (12)	0.0164 (3)	
C5	0.12805 (8)	0.7705 (3)	0.24651 (12)	0.0221 (4)	
H5A	0.1578	0.8947	0.2516	0.027*	
H5B	0.1087	0.7716	0.2933	0.027*	
C6	0.06556 (8)	0.3977 (3)	0.15837 (14)	0.0248 (4)	
H6A	0.0312	0.4272	0.0930	0.037*	
H6B	0.0514	0.4067	0.2141	0.037*	
H6C	0.0828	0.2300	0.1577	0.037*	

### Atomic displacement parameters (Å^2^)

**Table:**

	*U*^11^	*U*^22^	*U*^33^	*U*^12^	*U*^13^	*U*^23^
K1	0.01789 (19)	0.0171 (2)	0.01396 (19)	0.00072 (15)	0.00778 (15)	−0.00055 (14)
O1	0.0217 (6)	0.0208 (6)	0.0198 (6)	0.0039 (5)	0.0160 (5)	0.0020 (5)
O2	0.0228 (6)	0.0153 (6)	0.0153 (6)	0.0030 (5)	0.0095 (5)	0.0046 (5)
O3	0.0205 (6)	0.0232 (6)	0.0190 (6)	0.0044 (5)	0.0125 (5)	0.0050 (5)
O4	0.0167 (6)	0.0236 (6)	0.0204 (6)	0.0070 (5)	0.0108 (5)	0.0086 (5)
N1	0.0175 (7)	0.0143 (7)	0.0130 (7)	−0.0020 (6)	0.0076 (6)	−0.0014 (6)
C1	0.0142 (8)	0.0147 (8)	0.0156 (8)	0.0008 (6)	0.0080 (7)	0.0004 (6)
C2	0.0160 (9)	0.0148 (8)	0.0159 (8)	−0.0013 (7)	0.0065 (7)	−0.0038 (7)
C3	0.0198 (9)	0.0241 (9)	0.0201 (9)	0.0057 (8)	0.0082 (8)	0.0083 (7)
C4	0.0159 (8)	0.0185 (9)	0.0169 (9)	0.0066 (7)	0.0086 (7)	0.0053 (7)
C5	0.0261 (10)	0.0238 (9)	0.0207 (9)	0.0074 (7)	0.0138 (8)	0.0046 (7)
C6	0.0237 (10)	0.0247 (9)	0.0320 (10)	0.0009 (7)	0.0175 (9)	0.0046 (8)

### Geometric parameters (Å, °)

**Table:**

K1—O1^i^	2.7036 (10)	O4—C2	1.3591 (18)
K1—O2^ii^	2.7539 (11)	O4—C3	1.4447 (18)
K1—O3^i^	2.7988 (11)	N1—C1	1.3516 (19)
K1—O1	2.7994 (11)	N1—K1^iv^	3.2874 (12)
K1—O2	2.8896 (10)	C1—C2	1.451 (2)
K1—O3^iii^	2.8970 (12)	C1—C4	1.4917 (19)
K1—O1^iii^	2.9080 (11)	C1—K1^iv^	3.4260 (15)
K1—C5^ii^	3.0584 (16)	C2—K1^iv^	3.2747 (16)
K1—N1	3.2542 (13)	C3—H3A	0.9800
K1—C2^iii^	3.2747 (16)	C3—H3B	0.9800
K1—N1^iii^	3.2874 (12)	C3—H3C	0.9800
K1—C4^ii^	3.3355 (16)	C4—C5	1.325 (2)
O1—N1	1.2799 (14)	C4—C6	1.503 (2)
O1—K1^i^	2.7036 (10)	C4—K1^v^	3.3355 (16)
O1—K1^iv^	2.9081 (11)	C5—K1^v^	3.0583 (16)
O2—N1	1.2856 (15)	C5—H5A	0.9500
O2—K1^v^	2.7539 (11)	C5—H5B	0.9500
O3—C2	1.2219 (16)	C6—H6A	0.9800
O3—K1^i^	2.7989 (11)	C6—H6B	0.9800
O3—K1^iv^	2.8970 (12)	C6—H6C	0.9800
			
O1^i^—K1—O2^ii^	162.38 (3)	N1—K1—C4^ii^	93.33 (4)
O1^i^—K1—O3^i^	59.21 (3)	C2^iii^—K1—C4^ii^	145.11 (4)
O2^ii^—K1—O3^i^	106.36 (3)	N1^iii^—K1—C4^ii^	118.00 (3)
O1^i^—K1—O1	71.80 (3)	N1—O1—K1^i^	146.87 (9)
O2^ii^—K1—O1	117.16 (3)	N1—O1—K1	98.95 (7)
O3^i^—K1—O1	127.70 (3)	K1^i^—O1—K1	108.20 (3)
O1^i^—K1—O2	116.67 (3)	N1—O1—K1^iv^	95.49 (7)
O2^ii^—K1—O2	75.42 (2)	K1^i^—O1—K1^iv^	78.14 (3)
O3^i^—K1—O2	168.78 (3)	K1—O1—K1^iv^	135.94 (4)
O1—K1—O2	45.40 (3)	N1—O2—K1^v^	120.14 (8)
O1^i^—K1—O3^iii^	76.57 (3)	N1—O2—K1	94.53 (7)
O2^ii^—K1—O3^iii^	118.77 (3)	K1^v^—O2—K1	129.79 (4)
O3^i^—K1—O3^iii^	103.16 (3)	C2—O3—K1^i^	136.99 (9)
O1—K1—O3^iii^	80.73 (3)	C2—O3—K1^iv^	96.80 (9)
O2—K1—O3^iii^	85.11 (3)	K1^i^—O3—K1^iv^	76.84 (3)
O1^i^—K1—O1^iii^	101.86 (3)	C2—O4—C3	115.46 (12)
O2^ii^—K1—O1^iii^	82.21 (3)	O1—N1—O2	117.81 (11)
O3^i^—K1—O1^iii^	74.94 (3)	O1—N1—C1	123.09 (12)
O1—K1—O1^iii^	135.94 (4)	O2—N1—C1	119.10 (12)
O2—K1—O1^iii^	116.22 (3)	O1—N1—K1	58.18 (6)
O3^iii^—K1—O1^iii^	55.87 (3)	O2—N1—K1	62.27 (7)
O1^i^—K1—C5^ii^	96.14 (4)	C1—N1—K1	163.88 (10)
O2^ii^—K1—C5^ii^	72.78 (4)	O1—N1—K1^iv^	61.71 (6)
O3^i^—K1—C5^ii^	90.87 (4)	O2—N1—K1^iv^	127.10 (9)
O1—K1—C5^ii^	76.55 (4)	C1—N1—K1^iv^	84.21 (8)
O2—K1—C5^ii^	79.00 (4)	K1—N1—K1^iv^	107.96 (4)
O3^iii^—K1—C5^ii^	157.29 (4)	N1—C1—C2	120.36 (13)
O1^iii^—K1—C5^ii^	146.51 (4)	N1—C1—C4	117.74 (13)
O1^i^—K1—N1	93.48 (3)	C2—C1—C4	121.88 (13)
O2^ii^—K1—N1	98.04 (3)	N1—C1—K1^iv^	72.68 (8)
O3^i^—K1—N1	150.55 (3)	C2—C1—K1^iv^	71.72 (8)
O1—K1—N1	22.86 (3)	C4—C1—K1^iv^	129.38 (10)
O2—K1—N1	23.19 (3)	O3—C2—O4	121.61 (14)
O3^iii^—K1—N1	78.35 (3)	O3—C2—C1	129.29 (14)
O1^iii^—K1—N1	125.49 (3)	O4—C2—C1	109.10 (12)
C5^ii^—K1—N1	80.70 (4)	O3—C2—K1^iv^	61.45 (8)
O1^i^—K1—C2^iii^	97.99 (4)	O4—C2—K1^iv^	135.44 (9)
O2^ii^—K1—C2^iii^	98.10 (4)	C1—C2—K1^iv^	83.41 (9)
O3^i^—K1—C2^iii^	118.31 (4)	O4—C3—H3A	109.5
O1—K1—C2^iii^	83.79 (3)	O4—C3—H3B	109.5
O2—K1—C2^iii^	71.83 (3)	H3A—C3—H3B	109.5
O3^iii^—K1—C2^iii^	21.75 (3)	O4—C3—H3C	109.5
O1^iii^—K1—C2^iii^	53.27 (3)	H3A—C3—H3C	109.5
C5^ii^—K1—C2^iii^	150.79 (4)	H3B—C3—H3C	109.5
N1—K1—C2^iii^	73.06 (3)	C5—C4—C1	119.13 (14)
O1^i^—K1—N1^iii^	120.52 (3)	C5—C4—C6	123.51 (14)
O2^ii^—K1—N1^iii^	68.28 (3)	C1—C4—C6	117.33 (13)
O3^i^—K1—N1^iii^	96.40 (3)	C5—C4—K1^v^	66.48 (9)
O1—K1—N1^iii^	125.11 (3)	C1—C4—K1^v^	99.55 (9)
O2—K1—N1^iii^	94.52 (3)	C6—C4—K1^v^	105.93 (10)
O3^iii^—K1—N1^iii^	56.00 (3)	C4—C5—K1^v^	90.11 (10)
O1^iii^—K1—N1^iii^	22.80 (3)	C4—C5—H5A	120.0
C5^ii^—K1—N1^iii^	140.88 (4)	K1^v^—C5—H5A	88.2
N1—K1—N1^iii^	107.96 (4)	C4—C5—H5B	120.0
C2^iii^—K1—N1^iii^	43.50 (4)	K1^v^—C5—H5B	91.7
O1^i^—K1—C4^ii^	115.12 (4)	H5A—C5—H5B	120.0
O2^ii^—K1—C4^ii^	51.13 (3)	C4—C6—H6A	109.5
O3^i^—K1—C4^ii^	89.44 (4)	C4—C6—H6B	109.5
O1—K1—C4^ii^	95.85 (4)	H6A—C6—H6B	109.5
O2—K1—C4^ii^	83.16 (3)	C4—C6—H6C	109.5
O3^iii^—K1—C4^ii^	166.33 (3)	H6A—C6—H6C	109.5
O1^iii^—K1—C4^ii^	124.33 (4)	H6B—C6—H6C	109.5
C5^ii^—K1—C4^ii^	23.41 (4)		
			
O1^i^—K1—O1—N1	160.77 (10)	O1^iii^—K1—N1—O1	−125.49 (9)
O2^ii^—K1—O1—N1	−35.71 (9)	C5^ii^—K1—N1—O1	77.40 (8)
O3^i^—K1—O1—N1	−178.51 (7)	C2^iii^—K1—N1—O1	−115.56 (8)
O2—K1—O1—N1	−10.30 (7)	N1^iii^—K1—N1—O1	−141.94 (7)
O3^iii^—K1—O1—N1	81.97 (8)	C4^ii^—K1—N1—O1	97.17 (8)
O1^iii^—K1—O1—N1	72.42 (10)	O1^i^—K1—N1—O2	−179.41 (8)
C5^ii^—K1—O1—N1	−98.01 (8)	O2^ii^—K1—N1—O2	−12.78 (9)
C2^iii^—K1—O1—N1	60.23 (8)	O3^i^—K1—N1—O2	−158.75 (8)
N1^iii^—K1—O1—N1	45.79 (8)	O1—K1—N1—O2	−161.15 (13)
C4^ii^—K1—O1—N1	−84.68 (8)	O3^iii^—K1—N1—O2	105.08 (8)
O1^i^—K1—O1—K1^i^	0.0	O1^iii^—K1—N1—O2	73.36 (8)
O2^ii^—K1—O1—K1^i^	163.52 (4)	C5^ii^—K1—N1—O2	−83.74 (8)
O3^i^—K1—O1—K1^i^	20.72 (6)	C2^iii^—K1—N1—O2	83.29 (8)
O2—K1—O1—K1^i^	−171.07 (6)	N1^iii^—K1—N1—O2	56.91 (9)
O3^iii^—K1—O1—K1^i^	−78.80 (4)	C4^ii^—K1—N1—O2	−63.97 (8)
O1^iii^—K1—O1—K1^i^	−88.35 (6)	O1^i^—K1—N1—C1	81.4 (3)
C5^ii^—K1—O1—K1^i^	101.21 (5)	O2^ii^—K1—N1—C1	−112.0 (3)
N1—K1—O1—K1^i^	−160.77 (10)	O3^i^—K1—N1—C1	102.0 (3)
C2^iii^—K1—O1—K1^i^	−100.54 (4)	O1—K1—N1—C1	99.6 (4)
N1^iii^—K1—O1—K1^i^	−114.98 (4)	O2—K1—N1—C1	−99.2 (3)
C4^ii^—K1—O1—K1^i^	114.55 (4)	O3^iii^—K1—N1—C1	5.9 (3)
O1^i^—K1—O1—K1^iv^	−91.64 (6)	O1^iii^—K1—N1—C1	−25.9 (3)
O2^ii^—K1—O1—K1^iv^	71.88 (6)	C5^ii^—K1—N1—C1	177.0 (3)
O3^i^—K1—O1—K1^iv^	−70.93 (7)	C2^iii^—K1—N1—C1	−15.9 (3)
O2—K1—O1—K1^iv^	97.29 (7)	N1^iii^—K1—N1—C1	−42.3 (4)
O3^iii^—K1—O1—K1^iv^	−170.45 (6)	C4^ii^—K1—N1—C1	−163.2 (3)
O1^iii^—K1—O1—K1^iv^	180.0	O1^i^—K1—N1—K1^iv^	−56.32 (4)
C5^ii^—K1—O1—K1^iv^	9.57 (6)	O2^ii^—K1—N1—K1^iv^	110.31 (4)
N1—K1—O1—K1^iv^	107.58 (10)	O3^i^—K1—N1—K1^iv^	−35.66 (9)
C2^iii^—K1—O1—K1^iv^	167.82 (6)	O1—K1—N1—K1^iv^	−38.06 (7)
N1^iii^—K1—O1—K1^iv^	153.38 (4)	O2—K1—N1—K1^iv^	123.09 (9)
C4^ii^—K1—O1—K1^iv^	22.91 (6)	O3^iii^—K1—N1—K1^iv^	−131.83 (4)
O1^i^—K1—O2—N1	0.66 (9)	O1^iii^—K1—N1—K1^iv^	−163.55 (3)
O2^ii^—K1—O2—N1	166.92 (9)	C5^ii^—K1—N1—K1^iv^	39.34 (4)
O3^i^—K1—O2—N1	66.31 (19)	C2^iii^—K1—N1—K1^iv^	−153.62 (4)
O1—K1—O2—N1	10.16 (7)	N1^iii^—K1—N1—K1^iv^	180.0
O3^iii^—K1—O2—N1	−71.65 (8)	C4^ii^—K1—N1—K1^iv^	59.12 (4)
O1^iii^—K1—O2—N1	−119.59 (8)	O1—N1—C1—C2	5.1 (2)
C5^ii^—K1—O2—N1	92.06 (8)	O2—N1—C1—C2	−174.70 (13)
C2^iii^—K1—O2—N1	−89.27 (8)	K1—N1—C1—C2	−84.2 (4)
N1^iii^—K1—O2—N1	−126.92 (9)	K1^iv^—N1—C1—C2	55.71 (13)
C4^ii^—K1—O2—N1	115.38 (8)	O1—N1—C1—C4	−176.54 (12)
O1^i^—K1—O2—K1^v^	−135.53 (5)	O2—N1—C1—C4	3.7 (2)
O2^ii^—K1—O2—K1^v^	30.73 (5)	K1—N1—C1—C4	94.1 (3)
O3^i^—K1—O2—K1^v^	−69.88 (17)	K1^iv^—N1—C1—C4	−125.94 (12)
O1—K1—O2—K1^v^	−126.03 (7)	O1—N1—C1—K1^iv^	−50.60 (12)
O3^iii^—K1—O2—K1^v^	152.16 (5)	O2—N1—C1—K1^iv^	129.60 (12)
O1^iii^—K1—O2—K1^v^	104.22 (5)	K1—N1—C1—K1^iv^	−139.9 (3)
C5^ii^—K1—O2—K1^v^	−44.14 (6)	K1^i^—O3—C2—O4	−153.88 (10)
N1—K1—O2—K1^v^	−136.19 (11)	K1^iv^—O3—C2—O4	128.14 (13)
C2^iii^—K1—O2—K1^v^	134.54 (6)	K1^i^—O3—C2—C1	25.9 (3)
N1^iii^—K1—O2—K1^v^	96.89 (5)	K1^iv^—O3—C2—C1	−52.12 (17)
C4^ii^—K1—O2—K1^v^	−20.81 (5)	K1^i^—O3—C2—K1^iv^	77.98 (12)
K1^i^—O1—N1—O2	163.96 (11)	C3—O4—C2—O3	−2.9 (2)
K1—O1—N1—O2	18.87 (12)	C3—O4—C2—C1	177.36 (12)
K1^iv^—O1—N1—O2	−119.37 (11)	C3—O4—C2—K1^iv^	77.08 (17)
K1^i^—O1—N1—C1	−15.8 (2)	N1—C1—C2—O3	−11.9 (2)
K1—O1—N1—C1	−160.93 (12)	C4—C1—C2—O3	169.81 (15)
K1^iv^—O1—N1—C1	60.82 (14)	K1^iv^—C1—C2—O3	44.26 (15)
K1^i^—O1—N1—K1	145.09 (16)	N1—C1—C2—O4	167.86 (13)
K1^iv^—O1—N1—K1	−138.24 (6)	C4—C1—C2—O4	−10.4 (2)
K1^i^—O1—N1—K1^iv^	−76.67 (13)	K1^iv^—C1—C2—O4	−135.97 (11)
K1—O1—N1—K1^iv^	138.24 (6)	N1—C1—C2—K1^iv^	−56.16 (13)
K1^v^—O2—N1—O1	123.95 (10)	C4—C1—C2—K1^iv^	125.55 (13)
K1—O2—N1—O1	−18.09 (12)	N1—C1—C4—C5	101.20 (18)
K1^v^—O2—N1—C1	−56.24 (15)	C2—C1—C4—C5	−80.5 (2)
K1—O2—N1—C1	161.72 (11)	K1^iv^—C1—C4—C5	11.4 (2)
K1^v^—O2—N1—K1	142.04 (8)	N1—C1—C4—C6	−80.71 (18)
K1^v^—O2—N1—K1^iv^	49.79 (12)	C2—C1—C4—C6	97.62 (18)
K1—O2—N1—K1^iv^	−92.25 (8)	K1^iv^—C1—C4—C6	−170.51 (10)
O1^i^—K1—N1—O1	−18.26 (10)	N1—C1—C4—K1^v^	32.88 (14)
O2^ii^—K1—N1—O1	148.37 (8)	C2—C1—C4—K1^v^	−148.79 (12)
O3^i^—K1—N1—O1	2.40 (12)	K1^iv^—C1—C4—K1^v^	−56.92 (11)
O2—K1—N1—O1	161.15 (13)	C1—C4—C5—K1^v^	−87.97 (13)
O3^iii^—K1—N1—O1	−93.78 (8)	C6—C4—C5—K1^v^	94.06 (15)

Symmetry codes: (i) −*x*+1/2, −*y*+1/2, −*z*; (ii) −*x*+1/2, *y*−1/2, −*z*+1/2; (iii) *x*, *y*−1, *z*; (iv) *x*, *y*+1, *z*; (v) −*x*+1/2, *y*+1/2, −*z*+1/2.
